# Diversity of *cis*-regulatory elements associated with auxin response in *Arabidopsis thaliana*

**DOI:** 10.1093/jxb/erx254

**Published:** 2017-07-27

**Authors:** Pavel Cherenkov, Daria Novikova, Nadya Omelyanchuk, Victor Levitsky, Ivo Grosse, Dolf Weijers, Victoria Mironova

**Affiliations:** 1 Novosibirsk State University, Russian Federation; 2 Institute of Cytology and Genetics, Russian Federation; 3 Department of Agrotechnology and Food Sciences, Subdivision Biochemistry, Wageningen University and Research Center, The Netherlands; 4 Institute of Computer Science, Martin Luther University Halle-Wittenberg, Germany; 5 German Centre for Integrative Biodiversity Research (iDiv) Halle-Jena-Leipzig, Germany

**Keywords:** Auxin, AuxRE, ARF, bZIP, bHLH, transcriptional regulation, chromatin states, bioinformatics

## Abstract

The phytohormone auxin regulates virtually every developmental process in land plants. This regulation is mediated via de-repression of DNA-binding auxin response factors (ARFs). ARFs bind TGTC-containing auxin response *cis*-elements (AuxREs), but there is growing evidence that additional *cis*-elements occur in auxin-responsive regulatory regions. The repertoire of auxin-related *cis*-elements and their involvement in different modes of auxin response are not yet known. Here we analyze the enrichment of nucleotide hexamers in upstream regions of auxin-responsive genes associated with auxin up- or down-regulation, with early or late response, ARF-binding domains, and with different chromatin states. Intriguingly, hexamers potentially bound by basic helix–loop–helix (bHLH) and basic leucine zipper (bZIP) factors as well as a family of A/T-rich hexamers are more highly enriched in auxin-responsive regions than canonical TGTC-containing AuxREs. We classify and annotate the whole spectrum of enriched hexamers and discuss their patterns of enrichment related to different modes of auxin response.

## Teaser

Meta-analysis of auxin-responsive transcriptome data identified both known and novel *cis*-regulatory elements associated with specific response modes.

## Introduction

Auxin, a key plant hormone, regulates many processes via modulation of gene expression at the transcriptional level (reviewed in Paque and Weijers, 2016; Strader and Zhao, 2016; Weijers and Wagner, 2016). The signals regulating transcription are integrated at gene promoters, where transcription factors bind specific *cis*-regulatory elements, generally through direct interaction with a short DNA-binding site 6–12 bp in length (reviewed in Franco-Zorrilla and Solano, 2017).

A family of related AUXIN RESPONSE FACTORS (ARFs) mediates the primary transcriptional response to auxin (reviewed in Guilfoyle and Hagen, 2007). Different ARFs bind TGTC-containing *cis*-elements called AuxREs (Auxin Response Elements) presumably regardless of the cellular auxin level (Ulmasov *et al.*, 1997, 1999). When auxin levels are low, ARFs form heterodimers with their repressors, the Aux/IAA proteins (reviewed in Paque and Weijers, 2016; Strader and Zhao, 2016). Aux/IAAs inhibit ARF function by preventing their contact with the transcription initiation complex (Ito *et al.*, 2016) and/or through ensuring a repressive chromatin state mediated by their binding to TPL/TPR (TOPLESS and related) proteins that are hypothesized to recruit histone deacetylases (Long *et al.*, 2006; Szemenyei *et al.*, 2008). When auxin levels are high, Aux/IAA proteins are bound by TIR1/AFB auxin receptors and subsequently polyubiquitinated and degraded. Derepressed ARFs trigger the transcription changes, a process that may involve recruitment of SWI/SNF (SWITCH/SUCROSE NONFERMENTING) chromatin-remodeling ATPases (Wu *et al.*, 2015). The latter make the chromatin region more accessible for other transcription factors.

The complexity and diversity of auxin transcriptional response is provided by an abundance of family members in auxin receptors, Aux/IAAs, ARFs, and their cofactors (reviewed in Weijers and Wagner, 2016). ARFs can homodimerize on DNA (Ulmasov *et al.*, 1997; Guilfoyle *et al.*, 1998; Vernoux *et al.*, 2011; Boer *et al.*, 2014) and they also are able to heterodimerize with other transcription factors. Interactions were shown between ARF7 and MYB77 (Shin *et al.*, 2007); ARF8 and the basic helix–loop–helix (bHLH) factor BPEp (Varaud *et al.*, 2011); ARF6 with the bHLH (PIF4) and BZR1/BES1 factors (Oh *et al.*, 2014); ARF6/8 and MADS factor FUL (Ripoll *et al.*, 2015); and ARF3 with representatives of G2-like (KAN1 and KAN4) (Kelley *et al.*, 2012), bHLH (IND), Homeobox (RPL, KNAT1, and KNAT3), AP2 (BBM and PLT5), and TCP (TCP4 and TCP18) families (Simonini *et al.*, 2016). For some cofactors, the binding sites were found in close vicinity to the ARF-binding site, forming a composite AuxRE. In the case of ARF3, whose interaction with other transcription factors is directly influenced by auxin, auxin-dependent gene regulation may occur via the DNA-binding site of the partner proteins, and thus not require a core AuxRE (Simonini *et al.*, 2016).

Among coupling elements, the ABRE (abscisic acid response element) ACGTG(G/T)C (Choi *et al.*, 2000) was first described as a part of the composite auxin response element in the soybean GH3 promoter, and was shown to bind a bZIP (basic leucine zipper) transcription factor (Ulmasov *et al.*, 1995; Liu *et al.*, 1997). Direct interaction between bZIP and ARF transcription factors has not been shown, but Arabidopsis bZIP11-related transcription factors mediate auxin response via interaction with chromatin modulator ADA2b, a subunit of a histone acetylation complex (Weiste and Dröge-Laser, 2014). Whereas bZIP-binding sites are not sufficient to mediate auxin response themselves, they couple to AuxREs and enhance auxin-mediated transcription of a *GH3* gene in an auxin concentration-dependent manner (Ulmasov *et al.*, 1995; Weiste and Dröge-Laser, 2014).

Along with ABRE, plant bZIP transcription factors bind other ACGT-containing sites; among them, A-box (TACGTA), C-box (GACGTC), and G-box (CACGTG) sequences are bound more preferentially (Izawa *et al.*, 1993; reviewed in Foster *et al.*, 1994; Jakoby *et al.*, 2002). The G-box is highly enriched in ARF6-binding regions (Oh *et al.*, 2014), but it should be noted that the G-box is not restricted as a binding site for bZIPs. PIFs and MYCs of the bHLH family (Martínez-García *et al.*, 2000; Dombrecht *et al.*, 2007; Fernandez-Calvo *et al.*, 2011; Oh *et al.*, 2012; Kim *et al.*, 2016), AP2/ERF ABI4 (Zhang *et al.*, 2013), and BZR1/BES1 (Yu *et al.*, 2011; Oh *et al.*, 2012) transcription factors can all also bind this core. Transcription factors that interact with a common *cis*-element may compete (Zhang *et al.*, 2013) or co-operatively regulate (Oh *et al.*, 2012) the target gene.

Together with bZIP-binding sites, bHLH and the BZR1/BES1-binding HUD (Hormone Up at Dawn) motif CACATG (Walcher and Nemhauser, 2012; Oh *et al.*, 2014), MYB factor-binding site MRE (AACC) and MYB core (CNGTTR) (Shin *et al.*, 2007), and MADS-binding CArG box (CC[A/T]6GG) (Ripoll *et al.*, 2015) were shown to reside close to functional AuxREs. Thus, studying the footprints of transcription factor DNA binding might be an efficient way to indicate those factors involved in auxin response.

However, several analyses of auxin-responsive upstream regions have indicated the enrichment of additional non-TGTC-containing motifs (Pufky *et al.*, 2003; Doi *et al.*, 2008; Berendzen *et al.*, 2012; Mironova *et al.*, 2014). *Cis*-elements associated with early transcriptional activation attracted more attention than those for auxin inhibition and late response. To unravel the repertoire of auxin response elements and their association with up- or down-regulation, early or late response, we develop a bioinformatics approach for the systematic identification of hexamers enriched in auxin-responsive upstream regions. We apply that approach to a wide variety of publicly available transcriptome data sets on auxin response studies.

## Materials and methods

### Data sets

We collected all available data sets on exogenous auxin treatment from the GEO database. RNA-Seq was normalized with the TMM method from the ‘edgeR’ package (Robinson *et al.*, 2010; McCarthy *et al.*, 2012) and transformed with ‘voom’ (Law *et al.*, 2014) from the ‘limma’ package (Ritchie *et al.*, 2015; Phipson *et al.*, 2016*).* We processed all data from ATH1 microarrays by the ‘limma’ package or took pre-processed data when they were publicly available. For all RNA-Seq and microarray data, we applied the Benjamini–Yekutieli method (Benjamini and Yekutieli, 2001) to control the false discovery rate (FDR), and we report for each data set the subset of genes corresponding to the FDR of 0.05 and a fold change >3/2 or <2/3, which we call differentially expressed genes (DEGs). We applied this procedure to all available data sets and obtained 21 data sets containing at least 10 DEGs (Supplementary Table S1 at *JXB* online) (Armstrong *et al.*, 2004; Nemhauser *et al.*, 2004; Redman *et al.*, 2004; Okushima *et al.*, 2005; Delker *et al.*, 2010; De Rybel *et al.*, 2012; Bargmann *et al.*, 2013; Lewis *et al.*, 2013; Chaiwanon and Wang, 2015; Xuan *et al.*, 2015).

For annotating putative AuxREs in these 21 data sets, we compiled control and positive gene sets as follows: the control gene set contains 11 223 genes, not differentially expressed in any of the experiments from Supplementary Table S1. The positive gene set contains 2451 genes, differentially expressed in at least three data sets.

We obtained Arabidopsis genome sequence and annotation from TAIR10 and retrieved [–1500; +1] upstream regions relative to the transcription start site of 21 098 genes with a unique probe in the ATH1 microarray.

### Association of hexamers with auxin response

**Fig. 1.**
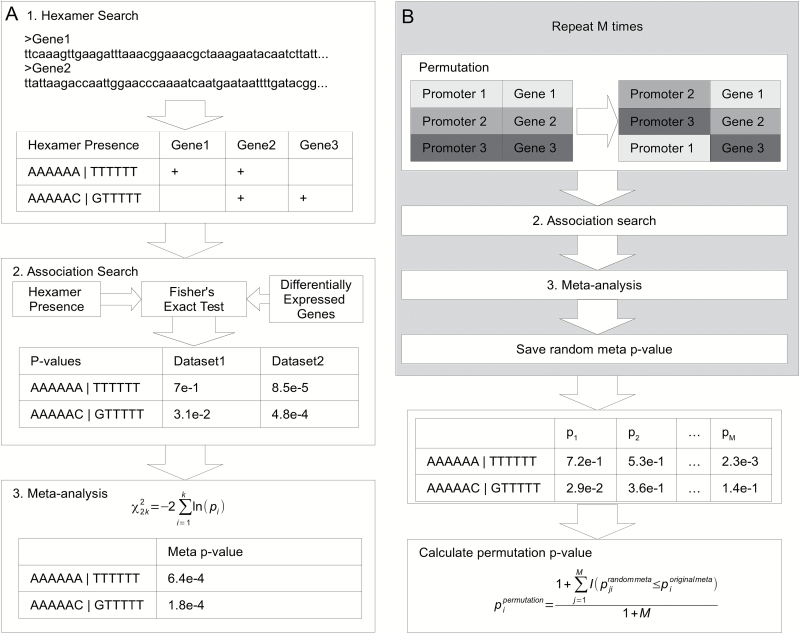
The pipeline for genome-wide association analysis for putative *cis*-elements associated with auxin response. (A) Three steps of the pipeline: (1) exhaustive hexamer search in the upstream regions; (2) analysis of association between the hexamer’s presence in the upstream region and auxin up- (down-) regulation of the gene; and (3) meta-analysis across all data sets. (B) Permutation test scheme, part of step (3).

The bioinformatics approach for predicting putative AuxREs is the further elaboration of our transcriptome meta-analysis method (Mironova *et al.*, 2014; Zemlyanskaya *et al.*, 2016). The approach consists of the following three-step procedure (Fig. 1).

In the first step, we searched for all possible hexamers in each of the upstream regions. Assuming the equivalence of hexamers on both DNA strands, we reduced the set of 4096 possible hexamers to 2080 non-redundant hexamers comprising 2016 complementary pairs and 64 palindromes. After this step, we obtained a list of hexamers per gene that occurred in its [–1500; +1] upstream region at least once.

In the second step, we analyzed for each hexamer and each data set the association of the presence of this hexamer in the gene upstream region and the gene status of being up- or down-regulated by auxin (Fig. 1A). We assess the significance of this association by the one-sided version of Fisher’s exact test (Table 1) for each data set, each hexamer, and each status. We combined for each hexamer the *P*-values for different data sets and different statuses using Fisher’s method:

**Table 1.** The contingency table for the analysis of associations between the presence of a hexamer in the upstream region of a gene ([–1500; +1] to transcription start site) and its auxin responsiveness

Genes	Auxin up- (down-) regulation		Totals
	Yes	No	
Hexamer (+)	A	B	A+B
Hexamer (–)	C	D	C+D
Totals	A+C	B+D	A+B+C+D

The data for every of 2080 hexamers in each whole-genome data set (Supplementary Table S1) were analyzed in the 2 × 2 contingency table using the Fisher’s exact test. The sum A+B+C+D is the number of all genes that have a unique probe on the ATH1 microarray platform.

$$\chi_{2k}^{2}=-2\sum_{i=1}^{k}\ln(p_{i})$$ (1)

where *P*_*i*_ is a *P*-value from a data set *i*, *k* is the number of data sets, and $\chi_{2k}^{2}$ is a chi-squared statistic with 2*k* degrees of freedom. The resulting meta *P*-value is the probability of obtaining a more extreme chi-squared statistic than the calculated one under the assumption that the *P*_*i*_ values are statistically independent. The hexamers with Bonferroni-corrected meta *P*-values <0.005 were selected for the next step.

In the third step, we determined the statistical significance of selected hexamers by a permutation test. In each permutation, we mixed promoters between genes so that each promoter was used exactly once (Fig. 1B). Then we performed the second and the third steps recording the meta *P*-values from Equation 1 for each permutation. After performing *M* permutations (*M=*1*e*+6) for each hexamer, we computed the permutation *P*-value by *P*=(*m*+1)/(*M*+1), where *m* is a number of recorded *P*-values not greater than the meta *P*-value.

We considered the association between the presence of the hexamer and the auxin responsiveness as significant with the permutation Bonferroni-corrected *P*-value <0.005.

### Comparison of identified hexamers with known *cis*-elements

We compared the detected hexamers with known *cis*-regulatory elements using TOMTOM (Gupta *et al.*, 2007) via DAP-seq (O’Malley *et al.*, 2016), PBM (Franco-Zorrilla *et al.*, 2014), and CIS-BP DNA databases (*Arabidopsis thaliana*). We considered the matches with an E-value <0.05 as significant. For hexamers without significant matches, we additionally screened the literature.

### Hexamer enrichment within ARF-binding regions

To study if the obtained hexamers are enriched within ARF-binding regions, we used available whole-genome data on ARF6 ChIP-Seq (Oh *et al.*, 2014; GSM1252254), ARF2, and ARF5 DAP-Seq (O’Malley *et al.*, 2016; GSM1925138, GSM1925826, and GSM1925827). For each hexamer, we compared two proportions via one-tailed Fisher test: (i) the number of positions a hexamer occupies across all 21 098 promoters [–1500; +1] relative to the total number of all possible positions; and (ii) the same across all peaks in a particular peak set. We adjusted *P*-values of enrichment with Bonferroni multiple testing correction. We considered over-representation as significant at a family-wise error rate (FWER) below 0.05.

### Hexamer enrichment in promoters without simple repeats

We applied RepeatMasker (Smit *et al.*, 2013–2015) with *-noint -s* parameters to all upstream regions for eliminating simple and tandem repeats from the initial set of 21 098 promoters. We searched for the hexamers within masked upstream regions and used the resulting lists through the pipeline (Fig. 1).

### Hexamer enrichment in promoters with different chromatin states

We used the data (Sequeira-Mendes *et al.*, 2014) on the distribution of nine chromatin states in the Arabidopsis genome to characterize [–1500; +1] upstream regions of auxin-responsive genes and putative AuxREs. First, we estimated if the chromatin states are uniformly distributed within upstream regions of auxin-responsive genes. For this, we performed the second and the third step of the association analysis (Fig. 1) with the chromatin state domains instead of hexamers.

Secondly, we tested if the hexamers were enriched in promoter segments associated with a specific chromatin state. For each hexamer, we compared two proportions via one-tailed Fisher’s exact test: (i) the number of positions which this hexamer occupies across all upstream regions of 21 098 genes relative to the total number of all possible positions; and (ii) the similar one across all promoter segments associated with a specific chromatin state.

Thirdly, we tested if the hexamers were enriched in the upstream regions located within specific chromatin states of auxin-responsive genes (positive set) relative to the same promoter segments of non-regulated genes (control set). For each hexamer, we compared two proportions via one-tailed Fisher’s exact test: (i) the number of positions this hexamer occupies in the promoter segments of a specific chromatin state within a positive gene set relative to the cumulative number of all possible positions in these segments; and (ii) the same proportion for the control gene set.

In each step, we adjusted *P*-values of enrichment with Bonferroni multiple testing correction, considering the association as significant at FWER <0.05.

## Results and Discussion

### Identification of auxin response *cis*-elements

To expand our knowledge on the scope of transcriptional regulation in auxin response, we aimed to detect putative AuxREs from meta-analysis of auxin-responsive transcriptome data sets without prior assumptions on the transcription factors binding these.

Dozens of auxin-related transcriptome data sets are publicly available in *A. thaliana* (Supplementary Table S1). Although the experiments were not designed to test the same hypothesis (they differ in dosage of applied auxin, duration of treatment, and tissue samples), systematic association of the same hexamer with auxin response in different experiments will diminish the probability that association of a hexamer is a random result. We developed a bioinformatics approach to search for putative AuxREs using many transcriptome inputs (see the Materials and methods; Fig. 1).

The procedure generated a list of hexamers (147 in total), which were substantially enriched in upstream regions of auxin-responsive genes (Supplementary Table S2). We considered these hexamers as putative novel AuxREs.

### A census of AuxREs

We found the canonical AuxRE core TGTCTC and its analog TGTCCC enriched in upstream regions of auxin-up-regulated genes, thus confirming previous findings (Ulmasov *et al.*, 1995; Xu *et al.*, 1997; Berendzen *et al.*, 2012). bZIP-binding ACGT-containing elements and the bHLH-binding HUD motif (CACATG), shown earlier as mediating auxin response (Ulmasov *et al.*, 1995; Liu *et al.*, 1997; Walcher and Nemhauser, 2012; Oh *et al.*, 2014; Weiste and Dröge-Laser, 2014), were also among those significantly associated with auxin response (Supplementary Table S2). These matches may be considered as an indication of the adequacy of the developed method.

Intriguingly, beyond the expected and known motifs, we found A/T-rich hexamers (not more than one G/C) to be the most abundant and the most significant in our search (Tables 2, 3). Two-thirds of the enriched hexamers were A/T rich, and they were not simple repeats, as we detected them even after filtering out these repeats from the upstream regions (see the Materials and methods). The relevance of TATA-box-like sequences to early auxin response was shown previously (Trenner *et al.*, 2017); however, only a part of the A/T-rich hexamers resembled TATA-box sequences (according to Yamamoto *et al.*, 2009) and peaked at the transcription start site (Supplementary Fig. S2). In Arabidopsis, many transcription factors bind *cis*-elements with more than five A/Ts in a row (O’Malley *et al.*, 2016); however, such an abundance might also be a sign of their involvement in epigenetic regulation (Roy *et al.*, 2016).

**Table 2.** Summary statistics on the number of detected auxin-associated *cis*-regulatory elements

	Early (≤2 h)	Late (>2 h)
Up	24 ^*a*^	78
Down	3	121
(i) Without A/T-rich hexamers		
Up	8^*a*^	16
Down	3	26
(ii) Specific in time of response and regulation		
Up	6^*a*^	18
Down	0	59
(iii) Enriched in ARF-binding regions		
Up	11^*a*^	25
Down	3	37

^*a*^ Including TGTCTC.

The total number of detected hexamers (top) and their classification by three characteristics: (i) belonging to non-A/T-rich elements; (ii) hexamers which were associated specifically with one of four gene groups (up/down and early/late); and (iii) hexamers significantly enriched in at least one peak set: ARF2-, ARF5- (O’Malley *et al.*, 2016), or ARF6-binding regions (Oh *et al.*, 2014).

For the source data see Supplementary Table S2.

**Table 3.** Overview on the statistical analysis results for predicted *cis*-regulatory elements associated with early auxin response

Hexamer	Transcriptome analysis, time of response				ChIP(DAP)-Seq data analysis			Description
	Early (≤2 h)		Late (>2 h)		Enrichment in ARF-binding regions			
	Up	Down	Up	Down	ARF2	ARF5	ARF6	
**Known and putative ARF-binding sites**								
TGTCTC	**				***	***	***	Classical AuxRE, ARF-binding core (Ulmasov *et al.*, 1997).
TGTCCC	***				***	***	***	AuxRE (Xu *et al.*, 1997; Weiste and Dröge- Laser, 2014).
GTCCCC	**				***	***	***	Putative AuxRE or TCP-binding core (Supplementary Fig. S1).
TGTGGG	***						***	
**bHLH- and BZR1/BES1-binding site**								
CACATG	***			***		***	***	HUD motif, enriched in ARF6-binding regions (Oh *et al.*, 2014).
**Putative MYB-binding site**								
GATAAG		***		**			***	MYB-binding core, I-box (Rose *et al.*, 1999) (Supplementary Fig. S1).
**Known and putative bZIP-binding sites**								
TACGTA	**		***	***			***	A-box, bound by bZIP factors (Izawa *et al.*, 1993).
ACGTAT	***		***	***			***	A-box-related
ACGTAG	**						***	
ACGTGT		***	***	***		**	***	G-box-related, ABRE, the binding sites for AREB/ABF factors (Yamaguchi-Shinozaki and Shinozaki, 2005).
ACGTGG		***		***			***	
**TATA-box-like, putative TBP-binding**								
TATAAA	***		***	***				Classical TATA-box (Heard *et al.*, 1993)
TATATA, ATATAT, ATATAC, ATATAG	***		***	***				TATA-like (Yamamoto *et al.*, 2009). Enriched near transcription start site (Supplementary Fig. S2).
ATATAA	***		***	***	**			
**Non-TATA-box A/T-rich**								
AACATT	**				***	***		Unknown A/T-rich, depleted near transcription start site (Supplementary Fig. S2).
CATAAT, GATTAA	***		***	***				
ACTATA, TATTAA	**		***	***				
ATTAGA, AAATAC	**		***					
CATATT	**			***			*	
CATTAT	**		***	***				
TAATTA	**		***	***				Putative ATHB-binding site (Supplementary Fig. S1).

In the meta-analysis we did not distinguish between the hexamer and its reverse complement. The data presented are only for the hexamers detected for the early responsive data sets (Supplementary Table S1); the complete data are given in Supplementary Table S2.

ChIP-Seq data for ARF6 were taken from (Oh *et al.*, 2014).

DAP-Seq data for ARF2 and ARF5 were taken from O’Malley *et al.*, 2016 (see the Materials and Methods).

*FWER <0.05; **FWER <0.01. ***FWER <0.001.

Analyzing the A/T-rich hexamers against the data on Arabidopsis transcription factor-binding sites generated by Franco-Zorrilla *et al.* (2014) and O’Malley *et al.* (2016) within the TOMTOM tool (Gupta *et al.*, 2007), we found 28 significant matches (E-value <0.05). Presumable A/T-rich binding sites for MYB-related (LCL1, LHY1, RVE1, EPR1, and others), G2-like (KAN4), AT-Hook (AHL20), B3 (VRN1, REM), and Homeobox (HAT1, 2, 5, 22; ATHB6, 13, 15, 18, 20, 23–24, 53; LMI1; PHV) transcription factors were detected here to be associated with the auxin response (Supplementary Table S3; Supplementary Fig. S1).

Among non-A/T-rich hexamers, TOMTOM found significant matches with MYB-binding sites for GATAAG and AGGGTT, a FUS3-binding site for CATGCA, TCP-binding sites for TGGGCC and GTCCCC, and a number of ACGT-containing sequences, which resemble the binding sites for several transcription factors families (bHLH, bZIP, NAC, and BZR1/BES) (Supplementary Fig. S1). A closer look at the ACGT-containing sequences and their auxin response pattern allowed identification of two major groups, G-box-related (CACGTG[G/T]) and A-box-related TACGTA[A/T][A/T] (Table 3).

What could over-representation of these hexamers in auxin-responsive regulatory regions mean? The identified hexamers could be the core sequences for transcription factor-binding sites mediating primary or secondary response. They could be the coupling hexamers for TGTC-containing AuxRE, constituting with it the composite element and bound by ARF partner transcription factors (Ulmasov *et al.*, 1995). Finally, some of the identified hexamers could influence formation of specific DNA conformations important for binding chromatin factors and thereby auxin response. In the next sections, we classify the identified hexamers to these groups.

### Putative AuxREs in early and late responses

Auxin-induced transcription occurs in temporal waves regulated by ARFs and their targets. The time of transcriptional response to auxin differs for various genes; for example, even among the early responding Aux/IAA gene family in Arabidopsis some genes respond within minutes while others only respond after 2 h (Abel *et al.*, 1995). To distinguish putative AuxREs mediating early and late responses, we performed a meta-analysis separately for the data sets with auxin treatment during <2 h (10 data sets) and the remainder (11 data sets). As a result, we identified 27 (24 up; 3 down) and 140 (78 up; 121 down) hexamers associated with early and late response, respectively (Table 2; Supplementary Table S2).

#### Early response

**Fig. 2.**
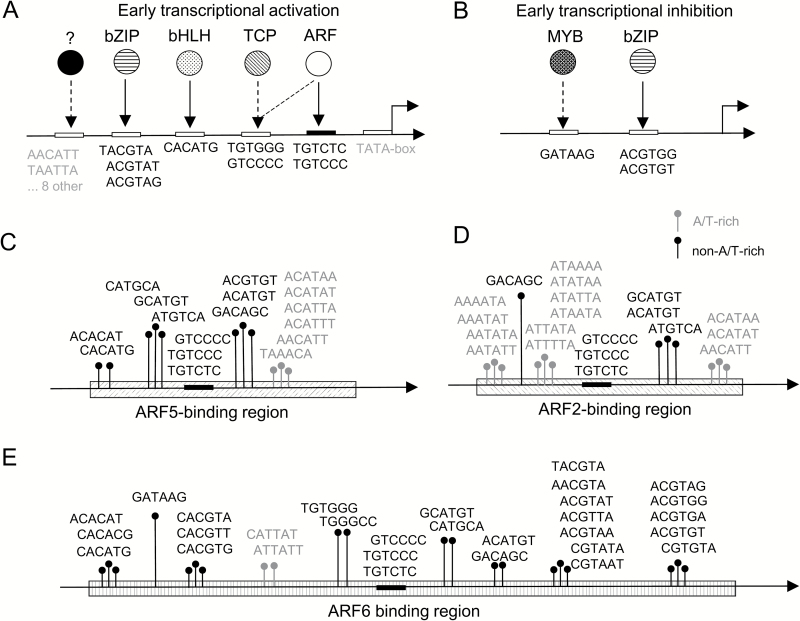
Scheme of the auxin response network reconstructed on the basis of predicted AuxREs. (A and B) *Cis*-regulatory elements conferring early auxin response (Table 3). (A) Activation of transcription. (B) Inhibition of transcription. (C–E) Potential coupling hexamers in composite AuxREs out of the whole list of auxin-associated *cis*-regulatory elements (Supplementary Table S2). The hexamers found significantly enriched within ARF5- (C); ARF2- (D), and ARF6-binding regions (E). The experimental data were taken from DAP-Seq analysis for ARF2 and ARF5 (O’Malley *et al.*, 2016) and ChIP-Seq analysis for ARF6 (Oh *et al.*, 2014). Pins were placed in random positions, as in this analysis we did not study the influence of orientation and relative position of the hexamers towards the TGTC-containing core.

Notably, for early activation and inhibition, we detected non-overlapping sets of hexamers (Fig. 2; Table 3).

The canonical AuxRE TGTCTC and its analog TGTCCC were specifically associated with activation of gene expression in early response to auxin (Tables 2, 23). The same association was found for TGTGGG and GTCCCC, which might be atypical ARF-binding AuxREs, or part of a TCP-binding site (Kosugi and Ohashi, 2002), as predicted by the TOMTOM tool for the latter (Supplementary Fig. S1). TATA-like hexamers (TATAAA, TATATA, ATATAA, ATATAT, ATATAC, and ATATAG), 10 non-TATA-like A/T-rich hexamers (depleted at the transcription start site; Supplementary Fig. S2), A-box-related putative bZIP-binding (TACGTA, ACGTAT, and ACGTAG), and bHLH-binding (CACATG) hexamers were also associated with early auxin-responsive transcriptional activation (Fig. 2A; Table 3).

No TGTC-containing elements were found to be associated with auxin-dependent down-regulation, which is consistent with findings published earlier (Mironova *et al.*, 2014; Zemlyanskaya *et al.*, 2016). However, we found G-box-related bZIP-binding (ACGTG[T/G]) and MYB-binding I-box (GATAAG; Supplementary Fig. S1) significantly associated with both early and late transcriptional repression (Fig. 2A).

#### Late response

There were a number of hexamers specifically associated with late up-regulation: BZR1-binding CACACG (He *et al.*, 2005), putative TCP-binding GGCCCA, and putative MYB-binding AACCCT (Supplementary Fig. S1; Supplementary Table S3) as well as a number of A/T-rich hexamers (Supplementary Table S2). However, the lists of hexamers associated with up- and down-regulation in late response significantly overlapped (Table 2); for instance, most of the A-box-related hexamers (including early response-related TACGTA and ACGTAT) and two G-box-related (TACGTG and ACGTGT) were found for both up-/down-regulation in late response (Supplementary Table S2). The remaining G-box-related hexamers, including the classical G-box hexamer CACGTG, were specifically associated with late repression. The abundance of potential bZIP-binding hexamers among detected putative AuxREs and their segregation between up- and down-regulation, early and late responses support the findings of bZIP factors as important modulators of auxin response (Weiste and Dröge-Laser, 2014; Ulmasov *et al.*, 1995).

The list of hexamers associated with late auxin down-regulation is almost twice longer than that for late up-regulation (Table 2). Besides ACGT-containing hexamers, EIN3-binding core ATGTA[T/C] (Kosugi and Ohashi, 2000) and a suite of A/T-rich elements are specific for late inhibition (Supplementary Table S2). The role of auxin in modulating EIN3 protein nuclear accumulation was shown earlier (He *et al.*, 2011); our data suggest that the EIN3-mediated secondary response to auxin also occurs systematically. A great abundance of A/T-rich hexamers within upstream regions of down-regulated genes suggests that they may function in converting chromatin into a packed inactive state (discussed below). However, among A/T-rich motifs specifically associated with the late inhibitory response, TOMTOM predicts the binding sites for Homeobox, MYB, and GATA factors (Supplementary Fig. S1).

### Do identified hexamers represent coupling elements to canonical AuxREs?

ARFs are known to heterodimerize with other transcription factors (Shin *et al.*, 2007; Varaud *et al.*, 2011; Oh *et al.*, 2014). We thus asked if the identified hexamers (Supplementary Table S2) are coupling hexamers to the canonical AuxRE. To test this hypothesis, we explored the available data on whole-genome ARF-binding sites mapping by ChIP-Seq (ARF6; Oh *et al.*, 2014) or DAP-Seq (ARF2 and ARF5; O’Malley *et al.*, 2016) methods. We estimated whether ARF-binding regions were enriched with the identified hexamers (Supplementary Table S2; see the Materials and methods). Early responsive TGTCTC and TGTCCC were found prominently within the binding regions of all three ARFs, supporting the adequacy of the applied method (Table 3; Supplementary Table S2). The non-A/T-rich hexamers associated with early auxin response were significantly enriched within ARF6-binding regions; in addition, bHLH-binding CACATG and bZIP-binding ACGTGT were linked to ARF5-binding regions (Fig. 2B; Table 3).

Most of the auxin late response non-A/T-rich hexamers were also found enriched within ARF-binding regions (Fig. 2B), with the exception of EIN3-binding ATGTA[T/C] (Kosugi and Ohashi, 2000), the putative MYB-binding site AACCCT, and seven other as yet unknown hexamers (Supplementary Table S2). These results support previously published data on enrichment of bZIP-, MYB-, and bHLH-binding core sequences in close proximity to TGTC-containing AuxREs (Shin *et al.*, 2007; Berendzen *et al.*, 2012; Walcher and Nemhauser, 2012; Oh *et al.*, 2014). However, we found a great variety of potential bZIP- and MYB-related hexamers which might be the binding sites for different homologs (Supplementary Table S3). We also predict FUS3- and TCP-binding sites to be the coupling elements in composite AuxREs.

A/T-rich hexamers showed a distinct pattern: most were scant in ARF-binding regions, except for two groups of hexamers (Fig. 2C, D). Hexamers of the first group are enriched within ARF2/5-binding regions; when aligned they gave an extended motif TAAACAT[A/T][A/T] (Fig. 2B), which significantly matched the YAB5-binding site in TOMTOM (E-value <0.05; Supplementary Fig. S1). A representative hexamer AACATT was specifically associated with early auxin up-regulation (Table 3).

The group of poly(A/T) hexamers is significantly enriched within ARF2-binding regions (Fig. 2B), which predicts that ARF2 has a partner with an A/T-rich transcription factor-binding site.

Available whole-genome maps (Oh *et al.*, 2014; O’Malley *et al.*, 2016) do not provide a complete picture for ARF-binding regions, as they were generated for only three transcription factors of the wide ARF family. Thus, we cannot exclude that the remaining hexamers adjoin binding sites for other ARFs, or operate in other conditions. The single hexamers also could be the binding sites for ARF interaction partners, that anchor ARFs on the DNA without requiring a canonical AuxRE.

### Association of AuxREs with different chromatin states

The abundance of non-TATA-box A/T-rich hexamers in the list of putative AuxREs (Supplementary Table S2) raised the question of whether these motifs function in building a specific chromatin landscape rather than in binding transcription factors. To test this hypothesis, we used the chromatin map generated by Sequeira-Mendes *et al.* (2014), where nine chromatin states were determined, each with distinctive properties in DNA sequence, CG methylation, nucleosome density, histone variants, and modifications. Upstream regions of genes are mainly composed of blocks of chromatin states 1 (core promoter), 2 (proximal promoter), 4 (distal promoter), and 5 (Polycomb-regulated repressed chromatin type). Chromatin states 3, 6, and 7 are more associated with intragenic regions, and states 8 and 9 correspond to heterochromatin.

First we tested whether the promoters of auxin-responsive genes have a bias in location within any of the chromatin states (see the Materials and methods). Upstream regions of both auxin up- and down-regulated genes appeared to be enriched with the chromatin in state 4 (FWER <0.001). Up-regulated genes additionally possess significantly higher portions of chromatin state 1 (FWER <0.001) and state 3 (FWER <0.05) in the upstream regions comparing non-responsive with auxin-responsive genes. Down-regulated genes were additionally enriched with chromatin states 2 (FWER <0.01) and 5 (FWER <0.001).

**Fig. 3.**
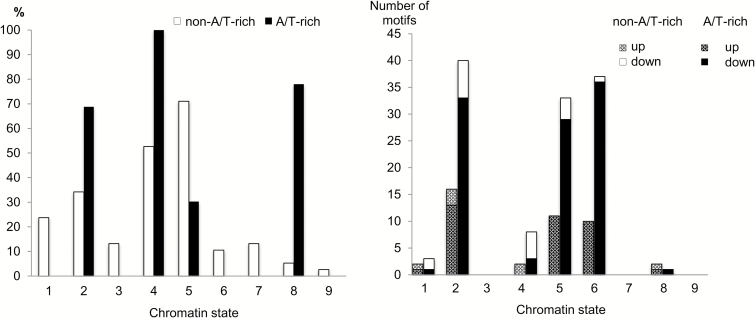
Putative AuxREs within chromatin context. (A) The portion of putative AuxREs that were found significantly enriched in the upstream regions associated with nine chromatin states (Sequeira-Mendes *et al.*, 2014). Significance was estimated via one-tailed Fisher’s exact test (see the Materials and methods). One hexamer can be enriched in more than one state. (B) The number of putative AuxREs specifically enriched in the chromatin state islands within the upstream regions of auxin-responsive genes relative to not auxin-responsive genes. Hexamers enriched in both up- and down-regulation are counted twice. A/T-rich hexamers are shown in gray.

Analyzing the distribution of putative AuxREs (Supplementary Table S4) over different chromatin states (see the Materials and methods), we found that all A/T-rich hexamers were significantly enriched in chromatin state 4, and large portions of putative AuxREs were also enriched in chromatin states 8 (72%), 2 (60 %), and 5 (26 %) (Fig. 3). Thus, A/T-rich hexamers might appear in our search results because of their abundance within specific chromatin states, which make up a notable part of auxin-responsive upstream regions. It was proposed that readers of short A/T-rich hexamers might restrain gene expression, for example by recruitment of DNA methylation or repressive histone marks (Quante and Bird, 2016).

**Fig. 4.**
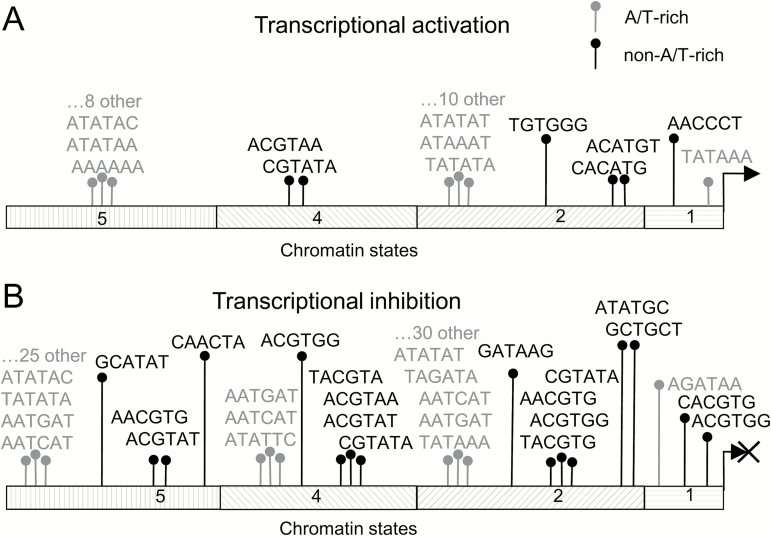
Putative AuxREs specifically enriched within chromatin states 1, 2, 4, and 5 of auxin-responsive genes. Core promoters tends to possess transcriptionally active chromatin state 1, proximal promoters usually belong to chromatin state 2, and distal promoters to state 4 or state 5 (Sequeira-Mendes *et al.*, 2014). (A) Association with transcriptional activation. (B) Association with transcriptional inhibition. A/T-rich hexamers are shown in gray. For details see Supplementary Table S4.

We next tested if putative AuxREs (Supplementary Table S4) are specifically distributed within certain chromatin domains of auxin-responsive upstream regions compared with non-regulated ones (see the Materials and methods). While we did not find any specific AuxRE association with chromatin state 3, it was the case for other chromatin states enriched in auxin-responsive genes (Figs 3B, 4).

Many A/T-rich putative AuxREs are specifically over-represented in chromatin states 2 and 5 of both auxin-activated and repressed genes, with the repressed genes showing a greater variety (Fig. 4; Supplementary Table S4). Most of them are unknown; however, TOMTOM predicts (E-value <0.05) a number of transcription factor-binding sites: Homeobox-related AAT[G/C]AT and KAN4-binding core ATATTC were significantly enriched within chromatin states 2, 4, and 5 of auxin-repressed genes. The TATA-box sequence TATAAA was enriched in core promoters (chromatin state 1) of only auxin-activated genes.

Negatively and positively auxin-responsive genes differed by non-A/T-rich hexamers associated with specific chromatin states (Fig. 4; Supplementary Table S4). All chromatin regions of auxin-inhibited genes were enriched with A-box- and G-box-related ACGT-containing hexamers; in auxin-activated genes, only chromatin state 4 was enriched with A-box-related ACGTA[A/T]A hexamers. In general, the chromatin landscape of upstream regions in auxin-inhibited genes was richer in specific hexamers than those of auxin-activated genes (Fig. 4B).

Interestingly TGTC-containing cores were not enriched in any chromatin state. Recently we showed that ethylene-responsive genes possess an EIN3-binding site within specific chromatin state 4 (Zemlyanskaya *et al.*, 2016); the present data predict a similar preference to bind DNA within specific chromatin domains for ARF partners, but not ARFs themselves.

The auxin transcriptional regulation machinery involves a number of chromatin-remodeling factors (Szemenyei *et al.*, 2008; Weiste and Dröge-Laser, 2014; Wu *et al.*, 2015); the variety of auxin-associated hexamers specifically enriched within a certain chromatin context suggests that there are as yet unknown players in epigenetic regulation of auxin response. While the transcription factors bound to non-A/T-rich motifs might recruit chromatin-remodeling complexes, A/T-rich motifs might facilitate binding with these complexes, or directly influence nucleosome positioning.

## Conclusions

In recent years, plant biologists have generated an enormous amount of whole-genome expression profiling data. Development of high-throughput sequencing technologies makes the growth of these big data even faster. Despite providing a challenge for comprehensive analysis, accumulation of the data also provides benefits when studying the intricate features which are highlighted under systematic analysis. A search for *cis*-regulatory elements mediating a complex response is an example, as the binding sites for major regulators should be over-represented in the promoters of DEGs in many data sets wherein the regulator is involved. Development of a bioinformatics method which detects systematically over-represented motifs over many related transcriptome data sets (Fig. 1), helped us to identify a comprehensive set of auxin-response elements, and most of them were novel. Our results predict the key players in early and late auxin response (Fig. 2A–B; Table 3), and expand our knowledge on potential ARF partners whose binding sites are enriched within ARF-binding regions (Fig. 2C–E). Application of the meta-analysis pipeline on the data for the chromatin landscape of the *A. thaliana* genome (Sequeira-Mendes *et al.*, 2014) suggested which *cis*-regulatory elements might be involved in epigenetic regulation of auxin response (Fig. 4). These results also highlight the benefits of employment of independent data in meta-analyses, which promise that new findings will appear from as yet understudied whole-genome data.

## Supplementary data

Supplementary data are available at *JXB* online.

Table S1. Transcriptome data sets used in the meta-analysis.

Table S2. Hexamers significantly associated with auxin response: meta- and permutation *P*-values; statistics for the hexamer over-representation in ARF-binding regions.

Table S3. Hexamers associated with auxin response: matches with the transcription factor-binding sites from DAP-seq (O’Malley *et al.*, 2016), PBM (Franco-Zorrilla *et al.*, 2014), and CIS-BP DNA databases (*A. thaliana*) found by the TOMTOM tool (Gupta *et al.*, 2007).

Table S4. Analysis of the hexamer enrichment within specific chromatin states (Sequeira-Mendes *et al.*, 2014) of auxin-responsive upstream regions.

Fig. S1. Significant matches (E-value <0.05) of putative AuxREs with the transcription factor-binding sites identified by TOMTOM (Gupta *et al.*, 2007).

Fig. S2. Distribution of A/T-rich putative AuxREs along the upstream regions of auxin-responsive genes.

## Supplementary Material

Supplementary Figures_S1_S2

Supplementary Tables_S1_S4

## Abbreviations:

AuxRE: auxin response element
DEG: differentially expressed gene.
