# Supplementary material for: Diversity of cis-regulatory elements associated with auxin response in Arabidopsis thaliana
Source: J Exp Bot. 2017 Jul 27;69(2):329–39. doi: 10.1093/jxb/erx254 (PMC5853796; doi:10.1093/jxb/erx254)
Supplement: Supplementary Figures_S1_S2 [file erx254_suppl_supplementary_figures_s1_s2.pdf]

Fig. S1. Significant matches (e−value<0.05) of auxin−associated cis−regulatory elements with transcription factor binding sites identified by Tomtom tool (Gupta et al., 2007).

For each element alignment of two logos are presented: a transcription factor binding site sequence (above) and a hexamer sequence (below).

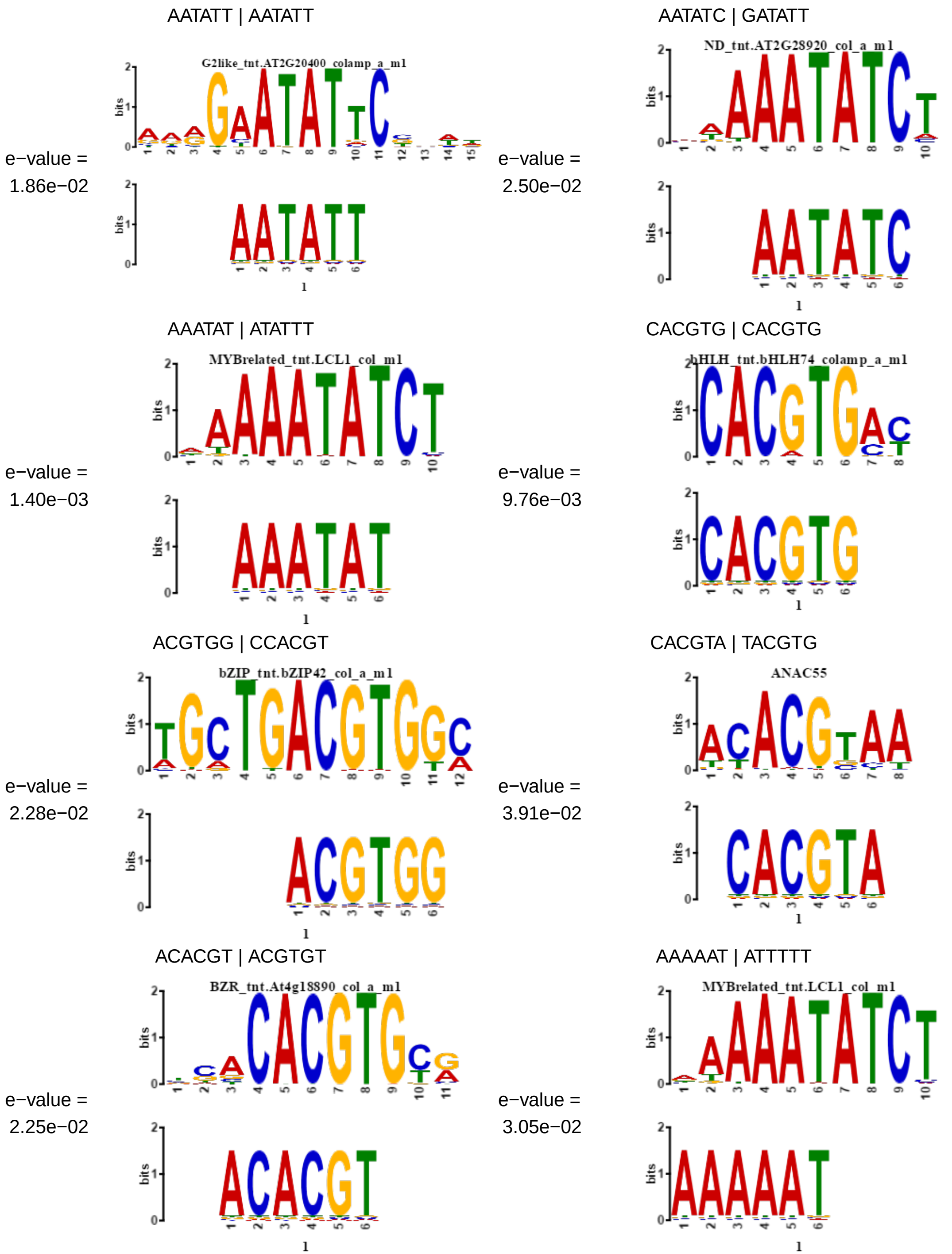

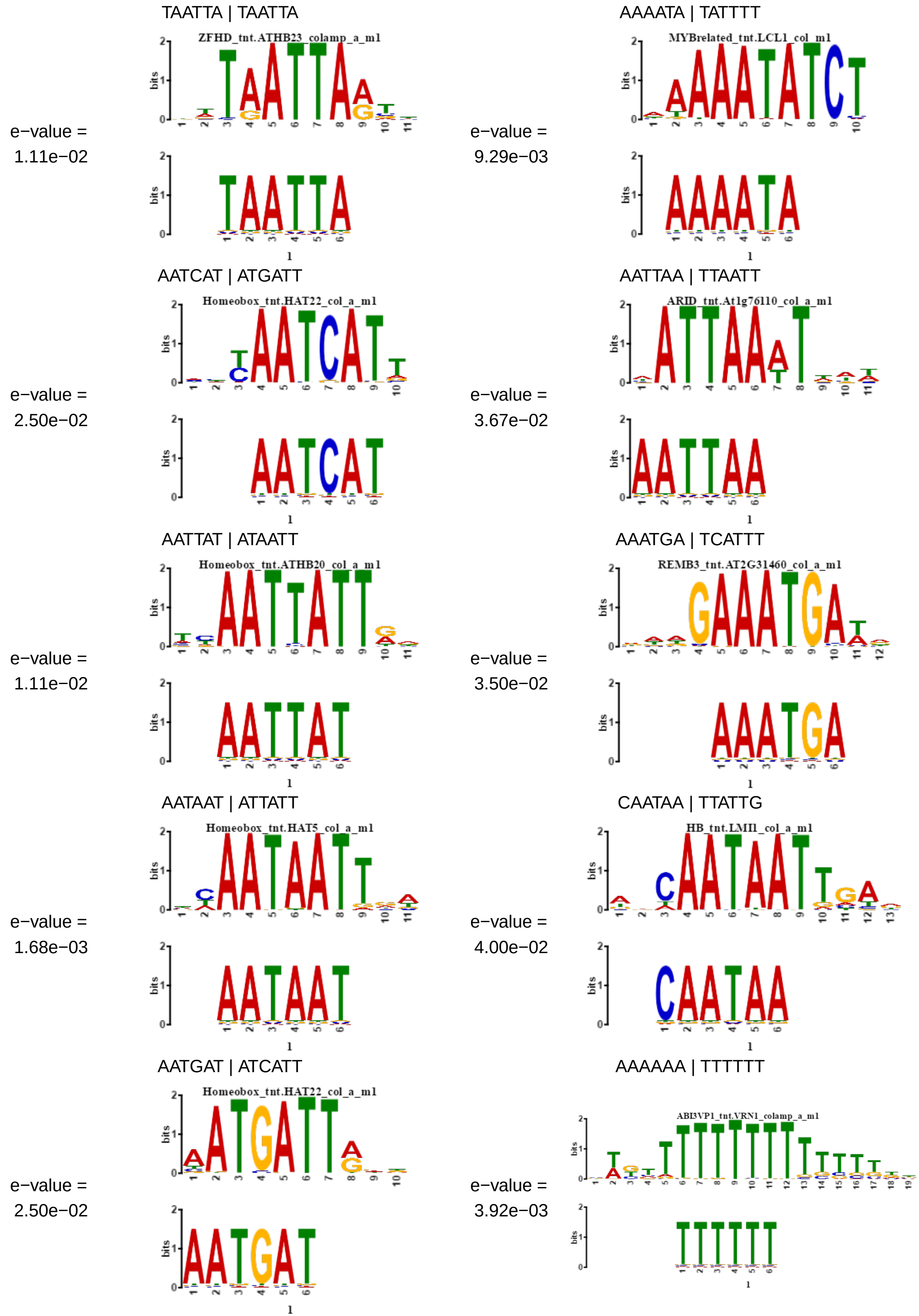
TAATTA | TAATTA
ZFHD_tnt.ATHB23_colamp_a_m1
e−value =
1.11e−02
AAAATA | TATTTT
MYBrelated_tnt.LCL1_col_a_m1
e−value =
9.29e−03
AATCAT | ATGATT
Homeobox_tnt.HAT22_col_a_m1
e−value =
2.50e−02
AATTAA | TTAATT
ARID_tnt.At1g76110_col_a_m1
e−value =
3.67e−02
AATTAT | ATAATT
Homeobox_tnt.ATHB20_col_a_m1
e−value =
1.11e−02
AAATGA | TCATTT
REMB3_tnt.AT2G31460_col_a_m1
e−value =
3.50e−02
AATAAT | ATTATT
Homeobox_tnt.HAT5_col_a_m1
e−value =
1.68e−03
CAATAA | TTATTG
HB_tnt.LMI1_col_a_m1
e−value =
4.00e−02
AATGAT | ATCATT
Homeobox_tnt.HAT22_col_a_m1
e−value =
2.50e−02
AAAAAA | TTTTTT
ABI3VP1_tnt.VRN1_colamp_a_m1
e−value =
3.92e−03
bits

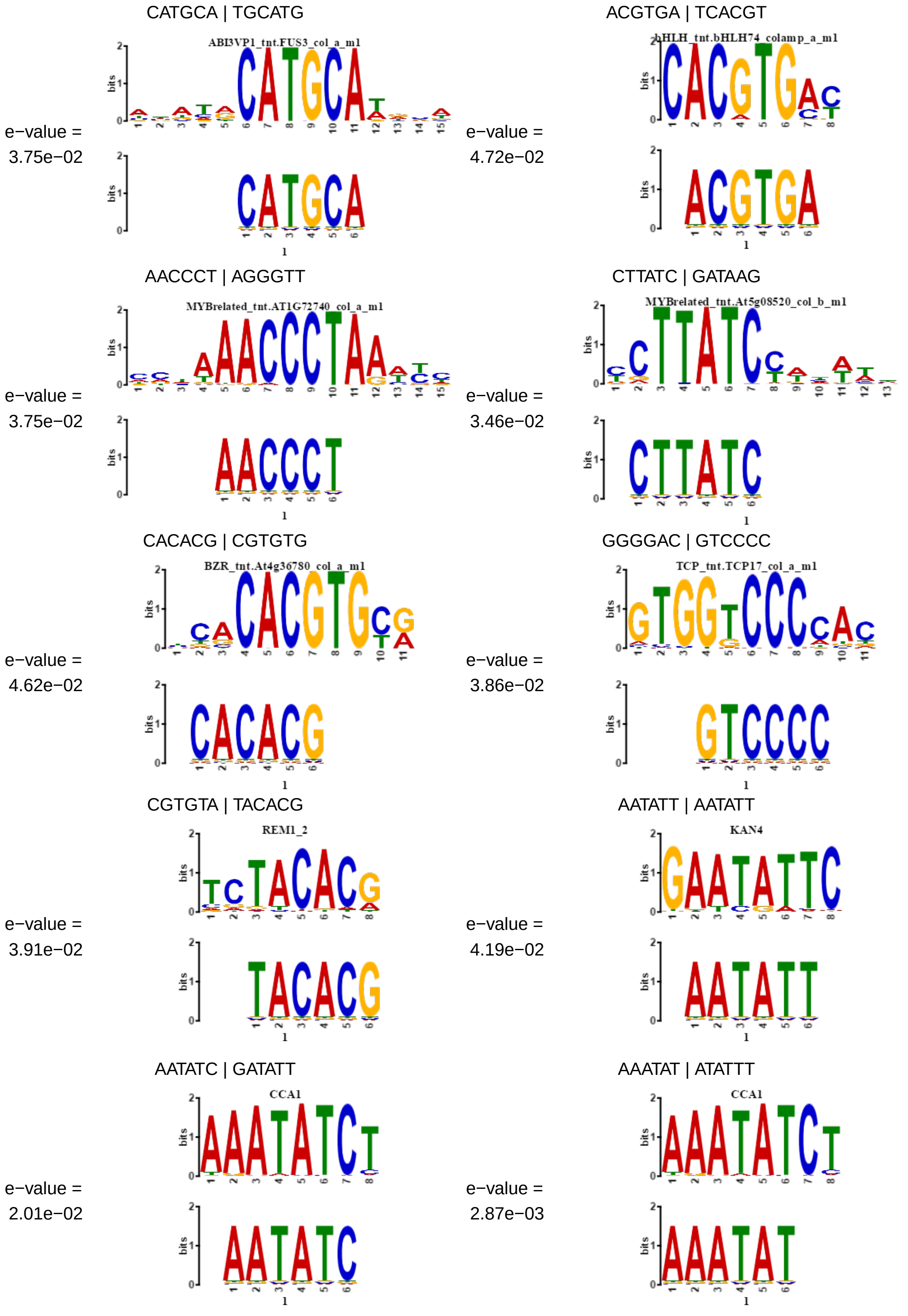
CATGCA | TGCATG
ABI3VP1_tnt.FUS3_col_a_m1
e−value =
3.75e−02
ACGTGA | TCACGT
bHLH_tnt.bHLH74_colamp_a_m1
e−value =
4.72e−02
AACCCT | AGGGTT
MYBrelated_tnt.AT1G72740_col_a_m1
e−value =
3.75e−02
CTTATC | GATAAG
MYBrelated_tnt.At5g08520_col_b_m1
e−value =
3.46e−02
CACACG | CGTGTG
BZR_tnt.At4g36780_col_a_m1
e−value =
4.62e−02
GGGGAC | GTCCCC
TCP_tnt.TCP17_col_a_m1
e−value =
3.86e−02
CGTGTA | TACACG
REM1_2
e−value =
3.91e−02
AATATT | AATATT
KAN4
e−value =
4.19e−02
AATATC | GATATT
CCA1
e−value =
2.01e−02
AAATAT | ATATTT
CCA1
e−value =
2.87e−03

CACGTG | CACGTG

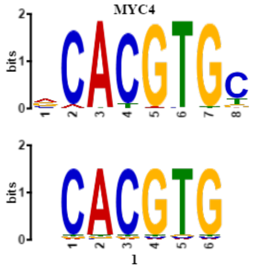

e−value = 2.40e−02

ACGTGG | CCACGT

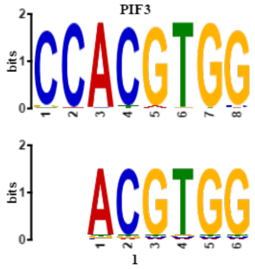

e−value = 2.40e−02

CACGTA | TACGTG

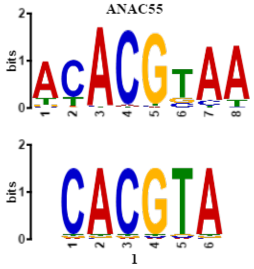

e−value = 3.91e−02

ACACGT | ACGTGT

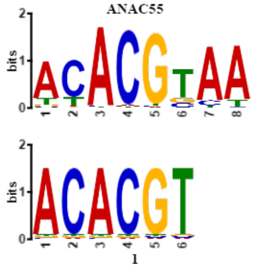

e−value = 3.91e−02

ATATTC | GAATAT

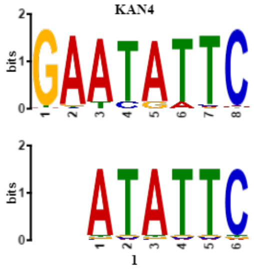

e−value = 2.01e−02

AACTAA | TTAGTT

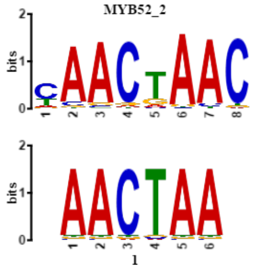

e−value = 2.01e−02

ATATAT | ATATAT

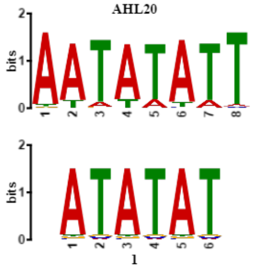

e−value = 4.19e−02

AATATA | TATATT

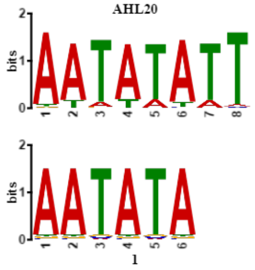

e−value = 3.15e−02

ACGTAA | TTACGT

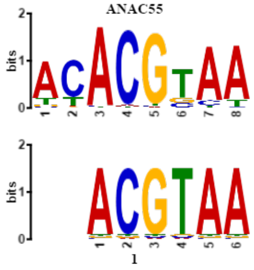

e−value = 2.13e−02

GGCCCA | TGGGCC

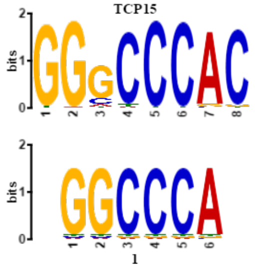

e−value = 2.54e−02

Fig. S2. Distribution of AT−rich hexamers identified as associated with early auxin response along the upstream regions in Arabidopsis genes.
The hexamers amount was calculated per each 100 nt in [−1500; +1] upstream regions of all genes (black line), auxin up−regulated (red line) and auxin down−regulated genes (blue line).

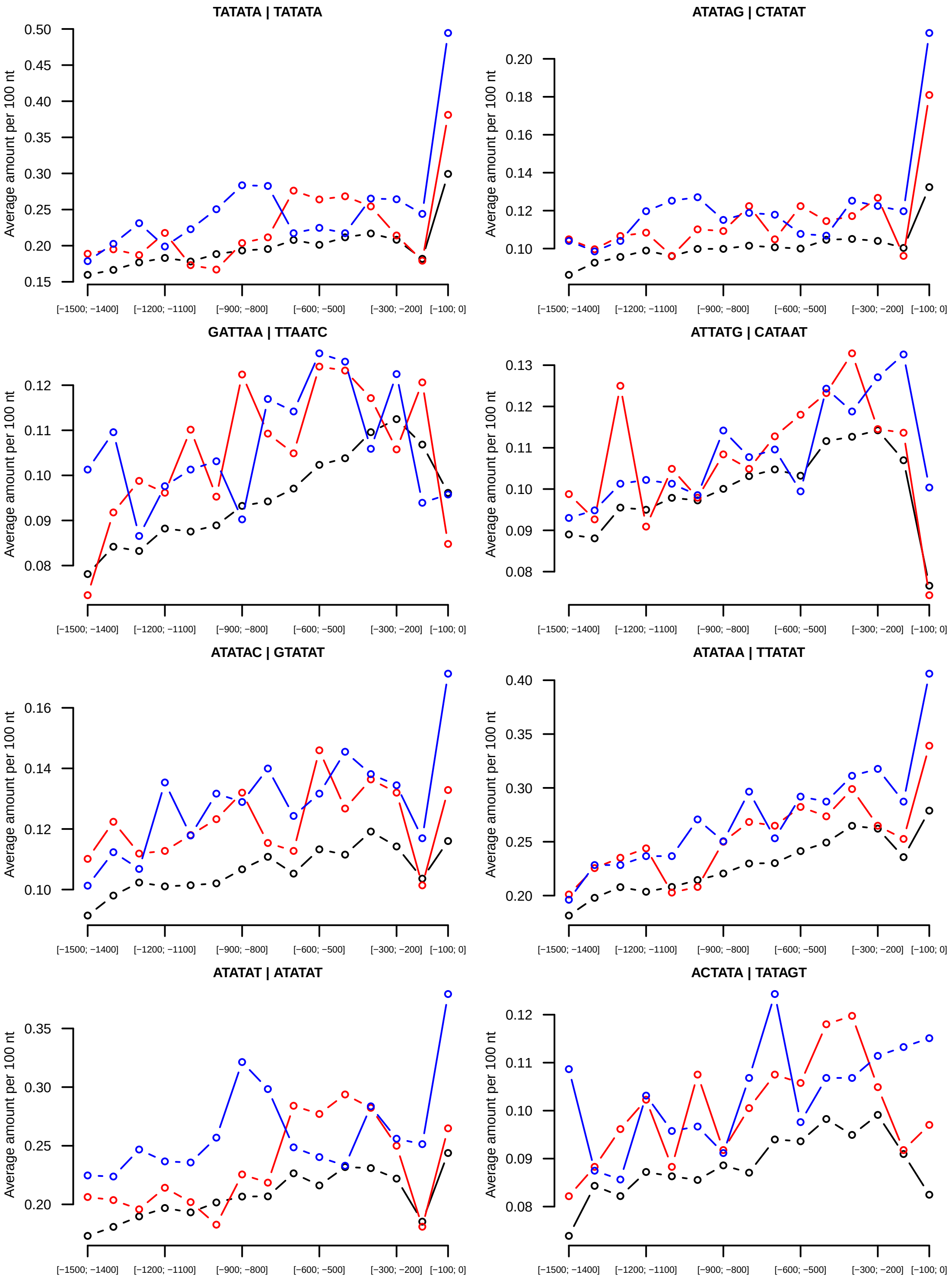

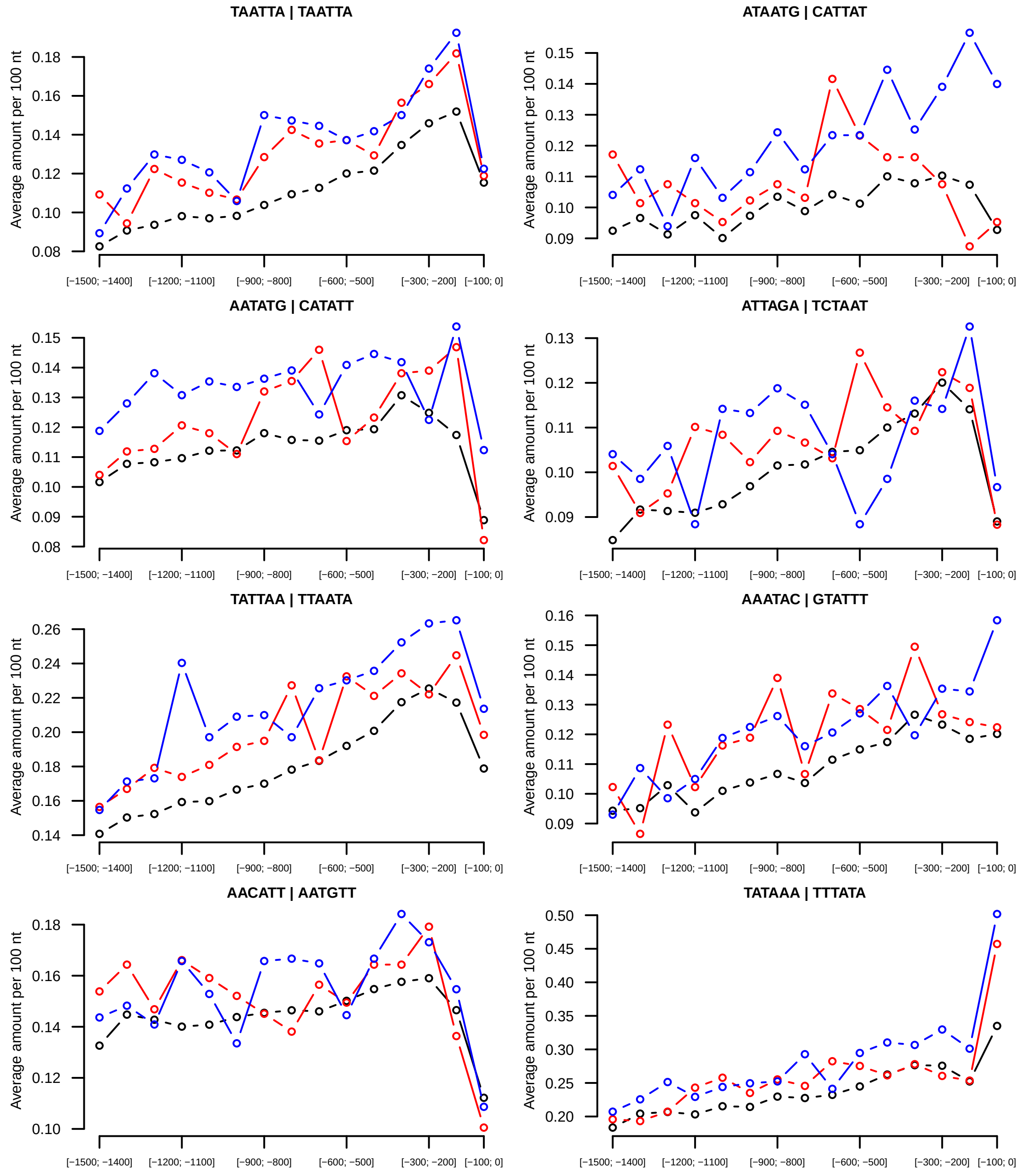
TAATTA | TAATTA
ATAATG | CATTAT
AATATG | CATATT
ATTAGA | TCTAAT
TATTAA | TTAATA
AAATAC | GTATTT
AACATT | AATGTT
TATAAA | TTTATA
Average amount per 100 nt
[−1500; −1400]
[−1200; −1100]
[−900; −800]
[−600; −500]
[−300; −200]
[−100; 0]
